# A Randomized, Open, Single-Administration, Cross-Over Study to Investigate the Acceptability, Swallowability, Palatability, and Preference of Different Oral Placebo Formulations in Patients with Multiple Sclerosis

**DOI:** 10.3390/pharmaceutics18030353

**Published:** 2026-03-12

**Authors:** Hans Martin Bosse, Kotryna Karosaite, Carolin Kloft, Melanie Schütte, Marc Pawlitzki, Philipp Albrecht, Sharmishtha Chauhan, Isabelle Gallou, Sebastien Chabaud, Tushar Sawai, Haripriya Tumuluri, Manfred Wargenau, Lucas-Sebastian Spitzhorn, Viviane Klingmann

**Affiliations:** 1 Department of General Pediatrics, Neonatology and Pediatric Cardiology, Medical Faculty, University Hospital Duesseldorf, Heinrich Heine University, 40225 Duesseldorf, Germany; hansmartin.bosse@med.uni-duesseldorf.de (H.M.B.); kotryna.karosaite@uni-duesseldorf.de (K.K.); carolin.kloft@med.uni-duesseldorf.de (C.K.); 2 Department of Neurology, Medical Faculty, University Hospital Duesseldorf, Heinrich Heine University, 40225 Duesseldorf, Germany; melanie.schuette@med.uni-duesseldorf.de (M.S.); marcguenter.pawlitzki@med.uni-duesseldorf.de (M.P.); philipp.albrecht@mariahilf.de (P.A.); 3 Department of Neurology, Maria Hilf Clinics, 41063 Mönchengladbach, Germany; 4 Novartis Healthcare Pvt. Ltd., Salarpuria-Sattva Knowledge City, Raidurg Madhapur, Ranga Reddy District, Hyderabad 500 080, India; sharmishtha.chauhan@novartis.com; 5 Novartis Pharma AG, Novartis Campus, 4056 Basel, Switzerland; isabelle_sylvie.gallou@novartis.com (I.G.); sebastien.chabaud@novartis.com (S.C.); haripriya.tumuluri@novartis.com (H.T.); 6 Novartis Healthcare Pvt. Ltd., Genome Valley, Turkapally, Lalgadi Malakpet, Shamirpet Mandal, Hyderabad 500 072, India; tushar.sawai@novartis.com; 7 M.A.R.C.O. GmbH & Co. KG, Institute for Clinical Research and Statistics, 40211 Duesseldorf, Germany; manfred.wargenau@marco-institut.de (M.W.); lucas.spitzhorn@marco-institut.de (L.-S.S.)

**Keywords:** acceptability, oral drug formulations, multiple sclerosis, palatability, swallowability, preference, drug administration

## Abstract

**Background:** There is a paucity of systematic investigations of the acceptability and preference of alternative oral drug formulations in multiple sclerosis (MS) patients. The use of appropriate oral dosage forms has the potential to circumvent challenges associated with the ingestion of tablets. **Objective:** This randomized, open, cross-over study aimed to investigate acceptability, swallowability, palatability, and preference of four oral placebo drug formulations of similar sizes/given volumes but different modes of ingestion (film-coated tablet, orodispersible tablet, orodispersible film, and gel) in MS patients. **Methods:** Acceptability was tested in two patient subgroups (32 participants each) of different MS disability levels (expanded disability status scale [EDSS] < 4 and ≥4). The primary endpoint was acceptability derived as a composite of swallowability (rated by investigator) and palatability (rated by participant). **Results:** The film-coated tablet showed the highest acceptability rates for EDSS < 4 and EDSS ≥ 4 (100.0%, 93.8%), followed by gel (81.3%, 68.8%). Acceptability rates for all formulations were consistently higher for EDSS < 4 compared to EDSS ≥ 4. Concerning the subjective assessment of palatability, the gel received the highest rate of positive ratings, but also was frequently judged as ‘Unpleasant’. Furthermore, the gel was ranked as the first or second choice as the most-preferred formulation, followed by the film-coated tablet. All formulations were considered safe in the study population. **Conclusions:** Film-coated tablets are well-suited for use in MS patients and gels may represent an interesting alternative for a certain subgroup of MS patients.

## 1. Introduction

Multiple sclerosis (MS) is the most common autoimmune disorder of the central nervous system in young adults, leading to disability and usually requiring life-long therapy [1]. Therefore, an increasing number of disease-modifying therapies have become available, several as oral tablets [2]. Additionally, many MS patients take oral medication for symptomatic treatment. However, especially in more severe cases, dysphagia and, as such, a risk of aspiration can occur [3,4], making oral tablet administration problematic. Such patients may favor other modes of oral applications like gels or orodispersible films/tablets.

The present study was conducted in view of the paucity of systematic investigations of the acceptability and preference of alternative oral drug formulations in MS patients. The use of appropriate oral dosage forms has the potential to circumvent challenges associated with the ingestion of tablets. This, in turn, can enhance patient compliance, which directly impacts the efficacy of the treatment regimen.

The European Medicines Agency (EMA) has identified a plethora of factors that may influence the acceptability of oral dosage forms in the pediatric population, such as swallowability and palatability [5]. Since these criteria may also be suitable for the adult patient population, this study applied a methodology of assessing the acceptability of an oral dosage form via a ‘composite endpoint’ based on swallowability and palatability. This method has been developed and validated in children aged newborn to <18 years [6,7,8].

## 2. Materials and Methods

### 2.1. Ethical Approval

This study received approval from the institutional ethics committee of the Medical Faculty of the Heinrich Heine University Duesseldorf (No. 2023-2439). The study was executed in accordance with the ethical principles originating from the Declaration of Helsinki [9] and is consistent with applicable Good Clinical Practice [10] regulatory requirements. Independent monitoring, data management, and statistical analysis of this study were performed by M.A.R.C.O. GmbH & Co. KG, Duesseldorf, Germany. German Clinical Trials Register: ID: DRKS00031786; https://drks.de/search/en/trial/DRKS00031786.

### 2.2. Study Population

**Figure 1:**
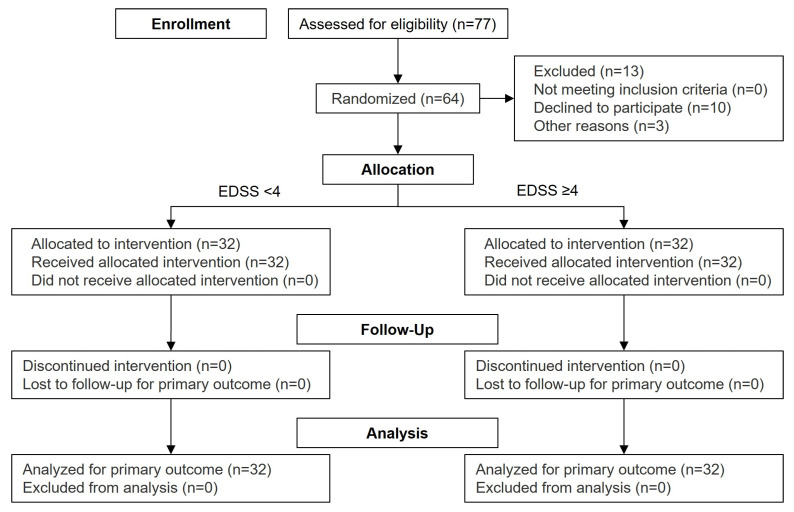
CONSORT flow diagram.

The participants (inpatients or outpatients) were recruited in the Department of Neurology of the University Hospital Duesseldorf, Germany. A total of 64 evaluable participants were included in the study, subdivided into two groups comprising 32 patients with either EDSS < 4 or ≥4 (see Figure 1). The EDSS grades the level of disability of the MS patients as assessed by levels of impairment of eight various functional systems, resulting in an overall score of 0 to 10, where a higher score represents a higher level of disability. The EDSS is thereby disproportionately influenced by the walking ability (EDSS < 4: able to walk more than 500 m) [11].

### 2.3. Inclusion and Exclusion Criteria

In this study, participants aged ≥18 years from both sexes who had diagnosed MS according to the 2017 McDonald criteria [12], including relapsing and progressive disease courses, were included. Participants had to be able to swallow the formulations, be willing and able to comply with the study procedures, and give written informed consent.

Exclusion criteria were a significant impairment of swallowing solids/liquids (dysphagia with relevant risk for aspiration) as assessed by the investigator and hypersensitivity to any of the ingredients present in any of the placebo formulations.

### 2.4. Formulations

**Figure 2:**
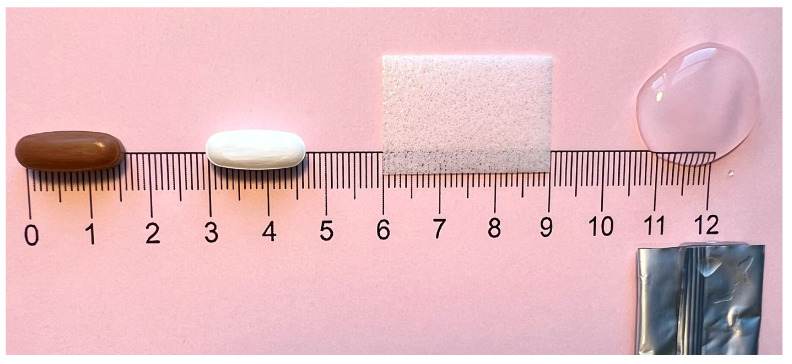
Formulations: From left to right: Film-coated tablet (16 mm), orodispersible tablet (16 mm), orodispersible film (20 × 30 mm), and gel (8 g).

Four oral placebo drug formulations (see Figure 2) were tested: oblong film-coated tablet (16 mm, coated with polyvinyl alcohol), orodispersible tablet (16 mm, silicified microcrystalline cellulose), orodispersible film (20 × 30 mm; polymer used: polyvinyl alcohol), and gel (8 g; polymer used: Xantham Gum). Except for the film-coated tablet (neutral taste), the formulations had similar intensities of orange flavor.

### 2.5. Study Design and Randomization

**Figure 3:**
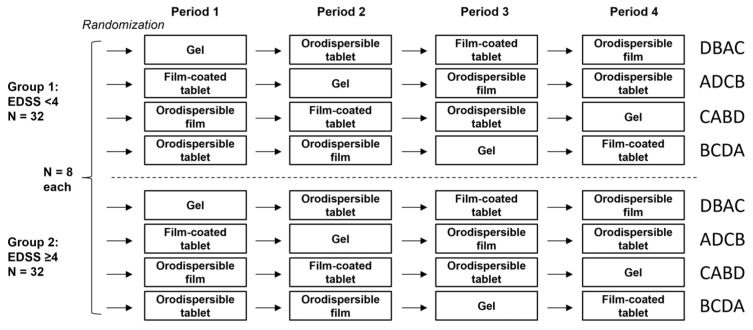
Study design.

This randomized open-label study was performed using a cross-over design (4 periods and 4 formulations) with stratification by EDSS groups (<4, ≥4). The four treatment sequences were chosen according to a Williams design [13], which is balanced for period and first-order carry-over effects. Randomization was done via a self-developed and validated SAS macro RANDOM (Figure 3).

### 2.6. Administration Procedure

All four formulations had to be taken by mouth with an approximately 5 min break between the two formulations, while a glass with water was available to support the deglutition process. The two tablet formulations were administered on a teaspoon, whereas the orodispersible film was placed into the mouth of the participant, and the gel was delivered in a pre-opened stick pack. The deglutition process was monitored by the qualified investigator, and the mouth was inspected shortly after swallowing the formulation. Swallowability and palatability were rated as described below.

### 2.7. Objectives

The primary objective of this study was to investigate the acceptability of a film-coated tablet, an orodispersible tablet, an orodispersible film, and a gel in patients with MS. As the size of the oblong film-coated tablet (16 mm) was considered potentially problematic for swallowing by MS patients, its acceptability in comparison to the three alternative oral drug formulations with similar sizes/volumes but different modes of ingestion was studied.

The secondary objectives of this study were to investigate the swallowability and palatability of these oral placebo formulations in patients with MS as assessed by the investigator as well as the participants. Furthermore, differences in these parameters between the two patient subgroups of differing MS disability levels were evaluated. Additionally, participants’ preferences for the different oral placebo formulations were investigated. Finally, the safety of the different formulations was investigated.

### 2.8. Endpoints

#### 2.8.1. Swallowability

Swallowability is the ability of the participant to swallow the dosage form as intended. Swallowability was assessed by the investigator using the scoring scheme shown in Table 1.

The swallowability of each formulation was also rated by the participant using the following five-point scale: 1: ‘Very good’; 2: ‘Good’; 3: ‘Neutral’; 4: ‘Bad’; 5: ‘Very bad’.

#### 2.8.2. Palatability

Palatability is the evaluation of the appearance, smell, taste, aftertaste, and mouth feeling of an oral medication. The participants assessed palatability using the following scale: 1: ‘Very pleasant’; 2: ‘Pleasant’; 3: ‘Neutral’; 4: ‘Unpleasant’; 5: ‘Very unpleasant’.

#### 2.8.3. Acceptability

Acceptability is the overall ability and willingness of the participant to take the dosage form as intended. It was derived as a ‘composite endpoint’ of swallowability (assessed by investigator) and palatability (assessed by participant), with the outcomes ‘High’, ‘Good’, ‘Low’, or ‘No’ (not acceptable) (Table 2).

#### 2.8.4. Preference

After having swallowed the last formulation in the last study period, the participants were asked to rank the different formulations from 1 (most preferred) to 4 (least preferred).

### 2.9. Determination of Sample Size

The formulations were compared in a pairwise manner in an exploratory sense. Thus, the confidence interval (CI) approach was applied for sample size consideration. Based on a sample size of 64, an observed 90% CI for a pairwise difference in the acceptability rates between the formulations of at least 10%-points (e.g., 90% vs. 80%) would not include zero.

### 2.10. Statistical Analysis

All statistical calculations were performed with the SAS^®^ software (SAS 9.4 Version, SAS-Institute, Cary, NC, USA). There was no missing data.

Age, gender and EDSS score are presented descriptively by stratum (EDSS < 4, ≥4), and overall. Percentages or means and standard deviations are presented as appropriate.

The primary endpoint of acceptability was analyzed as a binary variable on an intraindividual basis; i.e., the formulations were compared in a pairwise manner based on 2 × 2 contingency tables, applying the McNemar test. Two-sided 90% CIs were calculated for the difference in acceptability rates. Potential effects of EDSS group and formulations on the study endpoint and respective interaction were investigated by application of categorical data analysis with repeated measures using log-linear modeling. Frequency tables (counts, percentages) were provided by EDSS group and formulation. For the secondary endpoints, percentages were provided for all outcomes of swallowability and palatability by EDSS group and formulation. Analogously, results from participants’ preferences (ranking of formulations) were presented.

## 3. Results

### 3.1. Demographics and Baseline Characteristics

Participants from the EDSS < 4 group had a mean EDSS of 1.9 (±1.2) and a mean age of 39.8 (±12.7) years, whereas participants from the EDSS ≥ 4 group had a mean EDSS of 5.8 (±1.0) and a mean age of 50.7 (±11.9) years. Of the participants in the EDSS < 4 group, 68.8% were female and 31.2% male, whereas in the EDSS ≥ 4 group, 56.3% were female and 43.7% were male.

### 3.2. Primary Endpoint: Acceptability

All participants completed all four periods of swallowing the various formulations. As such, there was no missing data. In only one case, a participant refused to take one formulation. There, swallowability was evaluated with a score of five, ‘Refused to take’.

**Figure 4:**
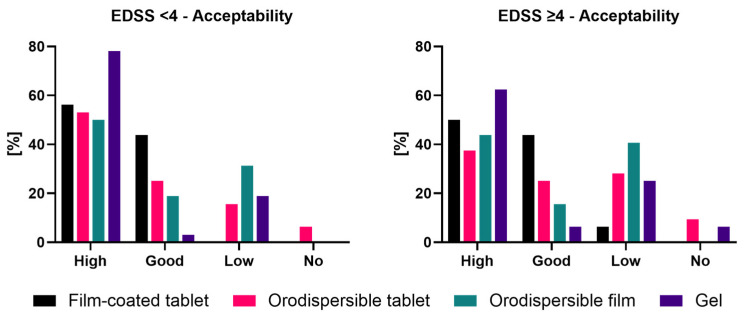
Acceptability as composite endpoint by EDSS group.

Results based on the acceptability evaluation criteria are shown in Figure 4 for both EDSS groups. Results for the composite endpoint expressed as binary outcome (‘Yes’ = ‘High’ or ‘Good’, ‘No’ = ‘Low’ or ‘No’) are shown in Table 3 and were the basis of the statistical comparisons between the film-coated tablet and the other three formulations.

EDSS < 4

Gel showed the highest rate for ‘High’ acceptability in the EDSS < 4 group (78.2%), whereas the film-coated tablet had the highest rate for combined ‘High’ and ‘Good’ acceptability (100.0% [56.2% ‘High’ and 43.8% ‘Good’]), followed by the gel (81.3% [78.2% ‘High’ and 3.1% ‘Good’]) and the orodispersible tablet (78.1% [53.1% ‘High’ and 25.0% ‘Good’]). The orodispersible film had the lowest combined acceptability rate (68.8% [50.0% ‘High’ and 18.8% ‘Good’]).

EDSS ≥ 4

Also, in the EDSS ≥ 4 group, the gel had the highest rate for ‘High’ acceptability (68.8%), but the film-coated tablet showed the highest rate for combined ‘High’ and ‘Good’ acceptability (93.8% [50.0% ‘High’ and 43.8% ‘Good’]), followed by the gel (68.8% [62.5% ‘High’ and 6.3% ‘Good’]) and the orodispersible tablet (62.5% [37.5% ‘High’ and 25.0% ‘Good’]). The orodispersible film had the lowest acceptability rate (59.4% [43.8% ‘High’ and 15.6% ‘Good’]).

The differences in acceptability rates between the film-coated tablet and each of the other formulations were significant in both EDSS groups (Table 3).

Potential effects of the EDSS group and formulations on the study endpoint and respective interaction were investigated by application of categorical data analysis with repeated measures using log-linear modeling of the frequencies presented in a contingency table. Statistical analysis demonstrated a formulation-specific effect on the composite acceptability rate; i.e., an overall difference between the various formulations was clearly demonstrated (*p*-value ≤ 0.0001). Furthermore, an EDSS-group-specific effect on the composite acceptability rate was statistically indicated (*p*-value = 0.0808 < 10%).

Acceptability rates for each formulation in the EDSS < 4 group were higher than the corresponding rates in the EDSS ≥ 4 group, with rate differences ranging from 6.2%-pts (film-coated tablet) to 15.6%-pts (orodispersible tablet).

Thus, since the EDSS group effect was consistently observed for each formulation, the EDSS-by-formulation interaction was not significant (*p* = 0.859), which especially allows for valid interpretation of the EDSS and formulation overall effects.

It appears noteworthy that acceptability rates significantly decreased over time, i.e., over the course of the four administrations, irrespective of the formulation. Acceptability rates (pooled over all four formulations) were estimated as 89.1%, 75.0%, 78.1%, and 64.1% for periods one, two, three, and four, respectively.

### 3.3. Secondary Endpoints: Swallowability, Palatability, Preference

#### 3.3.1. Swallowability

**Figure 5:**
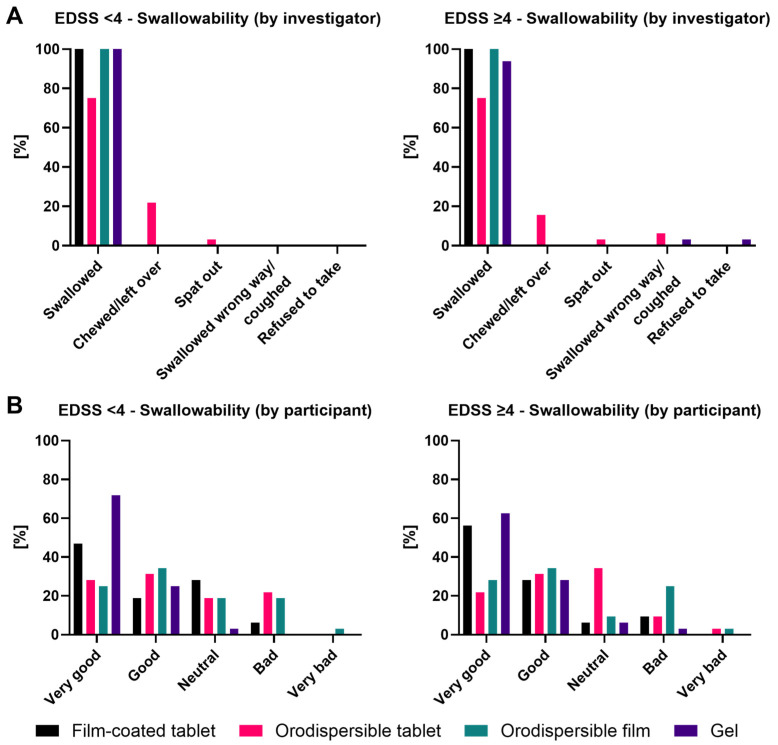
Swallowability as rated by investigator and participants by EDSS group. (**A**) Swallowability as rated by investigator; (**B**) swallowability as rated by participants.

Results of the swallowability assessment are displayed in Figure 5.

##### Swallowability by Investigator

EDSS < 4

Based on the observation of the investigator in the EDSS < 4 group, the film-coated tablet, the orodispersible film, and the gel were swallowed by 100.0% of the participants, whereas the orodispersible tablet was swallowed by only 75.0%. For this formulation, ‘Chewed/left over’ was documented in 21.9% and ‘Spat out’ in 3.1% of the cases (Figure 5A).

EDSS ≥ 4

Based on the observation of the investigator in the EDSS ≥ 4 group, the film-coated tablet and the orodispersible film were swallowed by 100.0%, the gel by 93.8%, and the orodispersible tablet by 75.0% of the participants. ‘Chewed/left over’ was documented in 15.6% of the cases for the orodispersible tablet. The orodispersible tablet was ‘Spat out’ in 3.1% of the cases. Deglutition problems, i.e., ‘Swallowed the wrong way/coughed’, were observed for the orodispersible tablet (6.3%) and the gel (3.1%). Only the gel was ‘Refused to take’, by 3.1% of the participants (Figure 5A).

##### Swallowability by Participant

EDSS < 4

Based on the participants’ self-assessment of swallowability in the EDSS < 4 group, the gel received the highest rate of ‘Very Good’/’Good’ ratings with 96.9% (71.9%/25.0%). The film-coated tablet received a 65.7% rate of ‘Very Good’/’Good’ ratings (46.9%/18.8%), the orodispersible tablet 59.4% (28.1%/31.3%) and the orodispersible film 59.4% (25.0%/34.4%) as well. A ‘Bad’ rating was noted for the orodispersible tablet by 21.9% of the participants, for the orodispersible film by 18.8%, and for the film-coated tablet by 6.3%. Only the orodispersible film was rated as ‘Very bad’, by 3.1% of the participants (Figure 5B).

EDSS ≥ 4

Also, in the EDSS ≥ 4 group, the gel received the highest rate of ‘Very Good’/‘Good’ ratings with 90.6% (62.5%/28.1%). The film-coated tablet received a 84.4% rate of ‘Very Good’/‘Good’ ratings (56.3%/28.1%), the orodispersible film 62.5% (28.1%/34.4%) and the orodispersible tablet 53.2% (21.9%/31.3%). A ‘Bad’ rating was noted for the orodispersible film by 25.0% of the participants, for the film-coated as well as for the orodispersible tablet by 9.4%, and for the gel by 3.1%. Only the orodispersible tablet and film were rated as ‘Very bad’, by 3.1% of the participants (Figure 5B).

#### 3.3.2. Palatability by Participant

**Figure 6:**
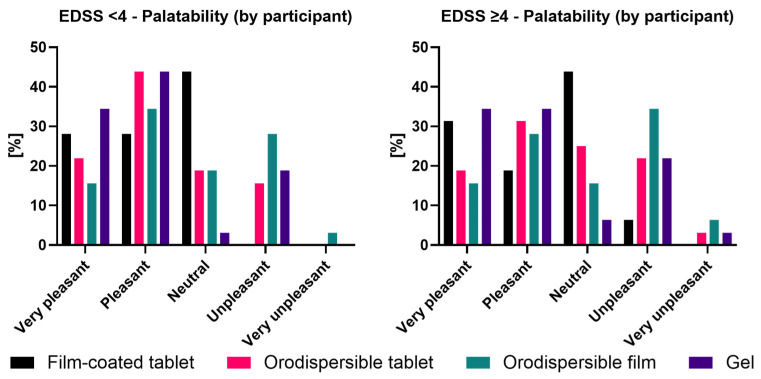
Palatability as rated by participants by EDSS group.

Results of the palatability assessment are graphically displayed by EDSS group in Figure 6.

EDSS < 4

Based on the participants’ self-assessment of palatability in the EDSS <4 group, the gel received the highest rate of ‘Very pleasant’/‘Pleasant’ ratings with 78.2% (34.4%/43.8%). The orodispersible tablet received a 65.7% rate of ‘Very pleasant’/‘Pleasant’ ratings (21.9%/43.8%), the film-coated tablet 56.2% (28.1%/28.1%), and the orodispersible film 50.0% (15.6%/34.4%). The film-coated tablet received the highest ‘Neutral’ ratings with 43.8%, the orodispersible tablet and film received this rating from 18.8%, and the gel from only 3.1% of participants. Although the gel had a high rate of positive evaluations, it also received ‘Unpleasant’ ratings from 18.8% (but no ‘Very unpleasant’ rating). The orodispersible tablet received ‘Unpleasant’ ratings from 15.6% but no ‘Very unpleasant’ rating. The orodispersible film received the highest rate of ‘Unpleasant’ ratings with 28.1% and was also rated as ‘Very unpleasant’ by 3.1%. The film-coated tablet was the only formulation that did not receive ‘Unpleasant’/‘Very unpleasant’ ratings (Figure 6).

EDSS ≥ 4

Also, in the EDSS ≥ 4 group, the gel received the highest rate of ‘Very pleasant’/‘Pleasant’ ratings with 68.8% (34.4%/34.4%). The film-coated tablet received a 52.1% rate of ‘Very pleasant’/‘Pleasant’ ratings (31.3%/18.8%), the orodispersible tablet received the same rate but with an inverted distribution (18.8%/31.3%), and the orodispersible film received 43.7% positive ratings (15.6%/28.1%). The film-coated tablet received the highest ‘Neutral’ ratings at 43.8%, the orodispersible tablet at 25.0%, the orodispersible film at 15.6%, and the gel at 6.3% of participants. Although the gel had a high rate of positive evaluations, it also received a ‘Unpleasant’/‘Very unpleasant’ rating from 25.0% (21.9/3.1%), which was equal to the orodispersible tablet. The orodispersible film received the highest negative ratings with 40.9% (34.4%/6.3%). The film-coated tablet received the lowest rate of ‘Unpleasant’ ratings with 6.3% and no ‘Very unpleasant’ rating (Figure 6).

#### 3.3.3. Participant’s Preference

**Figure 7:**
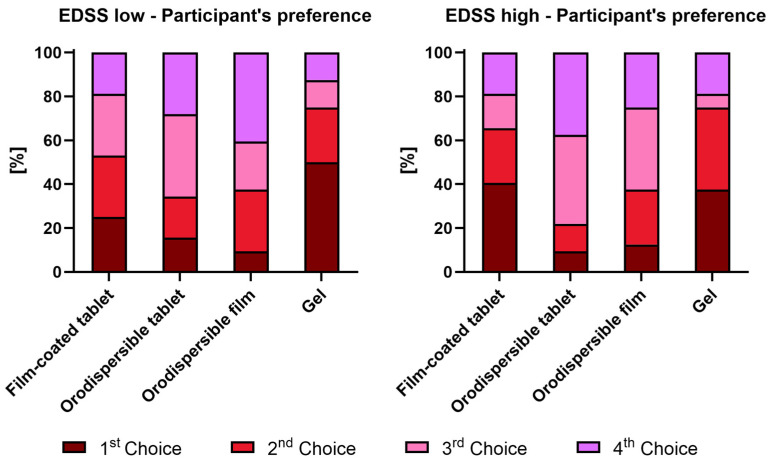
Participants’ preference (ranking of formulations) by EDSS groups.

A ranking of the formulations by the participants was done from their first choice (i.e., most preferred) to fourth choice (i.e., least preferred) (Figure 7).

EDSS < 4

Gel was selected as the first choice by 50.0% and as the second choice by 25.0% (in total 75%) of the participants from the EDSS < 4 group, whereas the film-coated tablet was selected as the first choice by only 25.0% and as the second choice by 28.1% (in total 53.1%) of the participants. The orodispersible tablet and the orodispersible film received the most ratings as the third or fourth choice by the participants (65.6% and 62.5%, respectively).

EDSS ≥ 4

Gel was selected as the first choice by 37.5% and as the second choice by 37.5% (in total 75%) of the participants from the EDSS ≥ 4 group. The film-coated tablet was selected as the first choice by 40.6% and as the second choice by 25.0% (in total 65.6%) of the participants. The orodispersible tablet (78.1%) and the orodispersible film (62.5%) received the most ratings as the third or fourth choice by the participants.

#### 3.3.4. Safety

Overall, ‘Swallowed the wrong way/coughed’ occurred in only two participants of the EDSS high group: it was observed for a male participant after intake of gel (in period two) and the orodispersible tablet (in period four), and for a female participant after intake of the orodispersible tablet (in period four). None of these events was evaluated as clinically significant.

## 4. Discussion

The aim of this study was to investigate the acceptability, swallowability, and palatability of and preference for different oral formulations with similar sizes/given volumes but different modes of ingestion in two MS patient groups (EDSS < 4, ≥4).

Overall, the participants in the EDSS < 4 group presented higher acceptability rates based on the composite endpoint compared to those in the EDSS ≥ 4 group, consistently for all formulations. This might be explained by the higher degree of impairment of MS patients in the EDSS ≥ 4 group, which could have affected the sensory sensitivity/perception during the swallowing process. This is underlined by the fact that all three patients who experienced deglutition problems were from the EDSS ≥ 4 group.

In both EDSS groups, the film-coated tablet significantly showed the highest acceptability rate (combined ‘High’ and ‘Good’ acceptability) compared to all other formulations. This is remarkable, as the swallowing of film-coated tablets with a size of 16 mm was considered potentially problematic for MS patients. The gel showed the highest rates of ‘High’ acceptability but lower ‘Good’ acceptability rates, resulting in the second highest acceptability rate in both EDSS groups.

Swallowability, as assessed by participants, showed the best ratings for gel, followed by the film-coated tablet in both EDSS groups. Palatability, as assessed by participants, showed the highest ‘Very pleasant/Pleasant’ evaluations for gel, but gel was also often rated negatively. The film-coated tablet indeed showed lower positive palatability evaluations than gel, but had higher ‘Neutral’ ratings and almost no negative ratings. This could be linked to the fact that the film-coated tablet did not contain added flavor, whereas the other dosage forms had orange flavor. This certainly is a limitation of this study. However, a sole advantageous or disadvantageous effect on the analysis results cannot be identified, since both positive and negative assessments of palatability frequently occurred (patients apparently either liked it very much or disliked it very much). Although no mouth-rinse with water was done in a standardized manner between taking the formulations to reduce any possible carry-over effects from one formulation to the other, participants were given sufficient time between the swallowing attempts.

When the participants were asked to rank the formulations, the gel was mainly ranked as the first or second choice in both EDSS groups. The film-coated tablet received the second most selections as the first or second choice. This result is remarkable since the acceptability as a composite endpoint showed a higher acceptability of the film-coated tablet compared to the gel. The percentage of participants who ranked gel as their first or second choice reflects the percentage of participants who rated the palatability of gel positively.

With regard to these findings, it is noteworthy that currently available MS treatments such as fingolimod, dimethyl fumarate, teriflunomide or siponimod are formulated as capsules or tablets [14,15,16,17]. Also, the newly developed Bruton’s tyrosine kinase inhibitor, tolebrutinib, is a film-coated tablet [18].

It is important to mention that only patients without diagnosed dysphagia were included in the study. However, dysphagia is a common complication of MS, and is especially apparent in patients with older age, longer disease duration and higher EDSS [3,19]. Depending on the study, 38–56% of patients with MS are described as facing dysphagia in various severities [4,20,21]. In such situations, gel or orodispersible formulations could be a better alternative than solid tablets. For example, in amyotrophic lateral sclerosis-induced dysphagia, the disease-modifying treatment Riluzole has also been developed as an oral suspension and oral film, since these formulations require less swallowing effort [22]. This could also be a way forward in MS treatment to omit the current practice of crushing tablets, which is associated with several limitations [23]. Future acceptability studies may include MS patients with dysphagia to collect data in a more real-world setting.

## 5. Conclusions

For this study, it can be concluded that film-coated tablets are well-suited for use in MS patients without diagnosed dysphagia. However, gels may represent an interesting alternative for a certain subgroup of MS patients. Importantly, all tested formulations were evaluated as safe in MS patients from the EDSS < 4 and ≥4 groups.

## Figures and Tables

**Table 1:** Scoring criteria for swallowability by the investigator.

Score	Observation
1	Swallowed: No chewing took place during deglutition and no residuals of the solid were found during oral inspection.
2	Chewed/left over: Chewing was observed before deglutition and/or the whole or parts of the solid were found in the mouth during oral inspection and/or left over on the spoon. ≥80% should be swallowed; if not, score 5 should be applied.
3	Spat out: No deglutition took place and the solid was no longer in the participant’s mouth.
4	Swallowed the wrong way/coughed: The solid was swallowed the wrong way or a cough was caused.
5	Refused to take: The participant did not allow the investigator to place the solid in the mouth.

**Table 2:** Scoring criteria for acceptability (composite endpoint).

Palatability Score (by Participant)	Swallowability Score (by Investigator)		
	1	2	≥3
Very pleasant/Pleasant	High	Good	No
Neutral	Good	Low	No
Very unpleasant/Unpleasant	Low	No	No

**Table 3:** Comparison of acceptability rates based on acceptability as composite endpoint by EDSS group.

Comparison		N	P(T) [%]	P(R) [%]	Rate Difference [%]	90% CI		*p*-Value
Test (T)	Reference (R)					Lower	Upper	
**EDSS < 4**								
Film-coated tablet	Orodispersible tablet	32	100.0	78.1	21.9	8.3	35.5	0.0082
	Orodispersible film	32	100.0	68.8	31.3	15.0	47.5	0.0016
	Gel	32	100.0	81.3	18.8	6.2	31.3	0.0143
**EDSS ≥ 4**								
Film-coated tablet	Orodispersible tablet	32	93.8	62.5	31.3	15.0	47.5	0.0016
	Orodispersible film	32	93.8	59.4	34.4	15.8	52.9	0.0023
	Gel	32	93.8	68.8	25.0	7.2	42.8	0.0209

CI = confidence interval; EDSS = expanded disability status scale; N = number of participants; *p* = percentage.

## Data Availability

The data presented in this study are available on request from the corresponding author.

## Abbreviations

The following abbreviations are used in this manuscript:

MS	Multiple sclerosis
EDSS	Expanded disability status scale
EMA	European Medicines Agency
